# From pixels to prognosis: unlocking the potential of deep learning in fibrotic lung disease imaging analysis

**DOI:** 10.1093/bjr/tqae108

**Published:** 2024-05-23

**Authors:** Sean R de la Orden Kett Morais, Federico N Felder, Simon L F Walsh

**Affiliations:** Faculty of Medicine, Imperial College, London, SW7 2AZ, United Kingdom; National Heart and Lung Institute, Imperial College, London, SW3 6LY, United Kingdom; National Heart and Lung Institute, Imperial College, London, SW3 6LY, United Kingdom

**Keywords:** high-resolution computed tomography, pulmonary fibrosis, idiopathic pulmonary fibrosis, imaging biomarkers, quantitative computed tomography, machine learning, survival

## Abstract

The licensing of antifibrotic therapy for fibrotic lung diseases, including idiopathic pulmonary fibrosis (IPF), has created an urgent need for reliable biomarkers to predict disease progression and treatment response. Some patients experience stable disease trajectories, while others deteriorate rapidly, making treatment decisions challenging. High-resolution chest CT has become crucial for diagnosis, but visual assessments by radiologists suffer from low reproducibility and high interobserver variability. To address these issues, computer-based image analysis, called quantitative CT, has emerged. However, many quantitative CT methods rely on human input for training, therefore potentially incorporating human error into computer training. Rapid advances in artificial intelligence, specifically deep learning, aim to overcome this limitation by enabling autonomous quantitative analysis. While promising, deep learning also presents challenges including the need to minimize algorithm biases, ensuring explainability, and addressing accessibility and ethical concerns. This review explores the development and application of deep learning in improving the imaging process for fibrotic lung disease.

## Introduction

The licensing of antifibrotic therapy for idiopathic pulmonary fibrosis (IPF) and its subsequent expansion to other progressive fibrotic lung diseases has underscored the critical need for dependable biomarkers for accurately predicting disease progression and treatment response.^1^ While some patients with fibrotic lung diseases experience stability, others undergo rapid deterioration. This variability poses challenges when determining the most appropriate treatment and the necessity for invasive procedures like surgical lung biopsy (SLB).^2–5^ The ability to predict disease behaviour based on baseline information would enable clinicians to identify individuals most likely to benefit from antifibrotic therapy, while also confidently holding back treatment in patients with inherently stable disease.

High-resolution CT of the chest (HRCT) is an essential diagnostic test in the initial evaluation of fibrotic lung disease and over the past decade, there has been a significant surge in interest in computer-based methods for the objective quantification of fibrotic lung diseases using HRCT. This increased interest has been motivated by the shortcomings of visual HRCT assessments by radiologists, including low reproducibility, high levels of interobserver variability, and limited sensitivity to small but clinically meaningful progression over short follow up periods.^2^^,^^6–8^

One such development in this field is quantitative CT (qCT) which involves training computers to quantify disease, voxel by voxel on HRCT. However, the requirement of human input to train the computer introduces the potential for human error being incorporated into the training procedure.^9^ The introduction of artificial intelligence, or more specifically, deep learning, into quantitative analysis aims to solve this problem. Deep learning has the advantage of carrying out quantitative analysis without regular human intervention. Deep learning offers the advantage of performing quantitative analysis autonomously, reducing the reliance on regular human intervention. However, the adoption of this technology is not without its challenges, including the need to minimize algorithm biases and ensure adequate algorithm explainability, particularly when computer-based predictions do not align with clinical decision-making. Furthermore, concerns exist regarding the accessibility of such technology and the establishment of robust ethical frameworks to govern its usage. This review will delve into the development and application of deep learning, shedding light on how it can significantly enhance the imaging process in fibrotic lung disease.

## Quantitative CT

The first step in moving away from subjective semi-quantitative evaluation and toward more objective quantitative CT was the application of HRCT histogram analysis which quantifies lung density per voxel of the HRCT image and represents it in a histogram.^6^ The kurtosis of the histogram describes the sharpness of its peak, whereas skewness is a measure of the lack of symmetry in the histogram. In the calculation of lung attenuation, kurtosis and skewness can be calculated and used as parameters to determine the presence, and extent of fibrosis.^10^^,^^11^ Typically, lung fibrosis causes increased mean lung attenuation, reduced kurtosis, and leftward skewness of the histogram.

In a study involving 144 patients with IPF, Best et al reported correlations between kurtosis and both physiologic decline and mortality.^11^ An important limitation this approach faces is in dealing with mixed disease patterns; it cannot distinguish between different HRCT patterns commonly observed in fibrotic lung disease patients. This early demonstration that computer-based voxel-wise analysis of HRCT data identifies correlations between lung characteristics and disease severity paved the way for more sophisticated methods. A recent study by Ash et al implemented local histogram-based quantification enabling separation of different HRCT patterns in a study of 46 IPF patients. This study reported strong correlations between visual assessments and objective histogram-based scores for disease extent and increasing mortality in patients with higher scores.^9^

### Quantitative lung fibrosis

Quantitative imaging analysis (QIA) is a software toolkit for HRCT image analysis comprising several computer-based quantification tools including quantitative lung fibrosis (QLF), quantitative honeycomb (QHC), ground glass (QGG), and composite interstitial lung disease (QILD) scores.^6^^,^^12^ QLF has demonstrated strong correlations with lung function measurements in ILD patients and was one of the first qCT tools to be implemented in a clinical treatment trial. In a study comparing cyclophosphamide to mycophenolate in 142 patients with scleroderma related ILD, Tashkin et al reported that QLF scores remained relatively unchanged in both treatment arms, while QILD scores showed a slight improvement in both the groups.^13^ The inclusion of QLF and QILD scores as secondary outcomes in clinical trials highlights the potential for computer-based imaging analysis tools to provide additional measures of disease progression alongside traditional lung physiology assessments, such as forced vital capacity (FVC).^14^^,^^15^

### CALIPER

Computer-aided lung informatics for pathology evaluation and ratings (CALIPER), developed by the Biomedical Imaging Resource Laboratory at the Mayo Clinic, leverages histogram signatures to characterize and quantify HRCT patterns of disease parenchymal. The system is trained using exemplar imaging data depicting these patterns, confirmed by expert radiologist consensus. CALIPER has demonstrated its efficacy in predicting outcomes across various ILDs.^6^ In a key study involving 283 patients with IPF, CALIPER-based HRCT scores were superior in predicting patient outcomes compared to traditional visual assessments of fibrosis.^16^ Building upon these findings, the authors explored a CALIPER-based staging model for mortality prediction which demonstrated improved prognostic discrimination when compared to the widely used GAP (gender-age-physiology) staging system in a bivariate analysis. In addition to fibrosis scores, CALIPER also introduced a novel HRCT parameter related to pulmonary vessels, referred to as “vessel-related structures.” When expressed as a percentage of the total lung volume, this parameter emerged as the most robust predictor of mortality among all CALIPER and visual HRCT pattern scores.^17^ Furthermore, this biomarker has undergone validation in various subsets of fibrotic lung diseases, including chronic hypersensitivity pneumonitis, connective tissue disease related ILD, and unclassifiable ILD, across multiple studies.^18–20^

### The adaptive multiple features method

**Figure 1.**
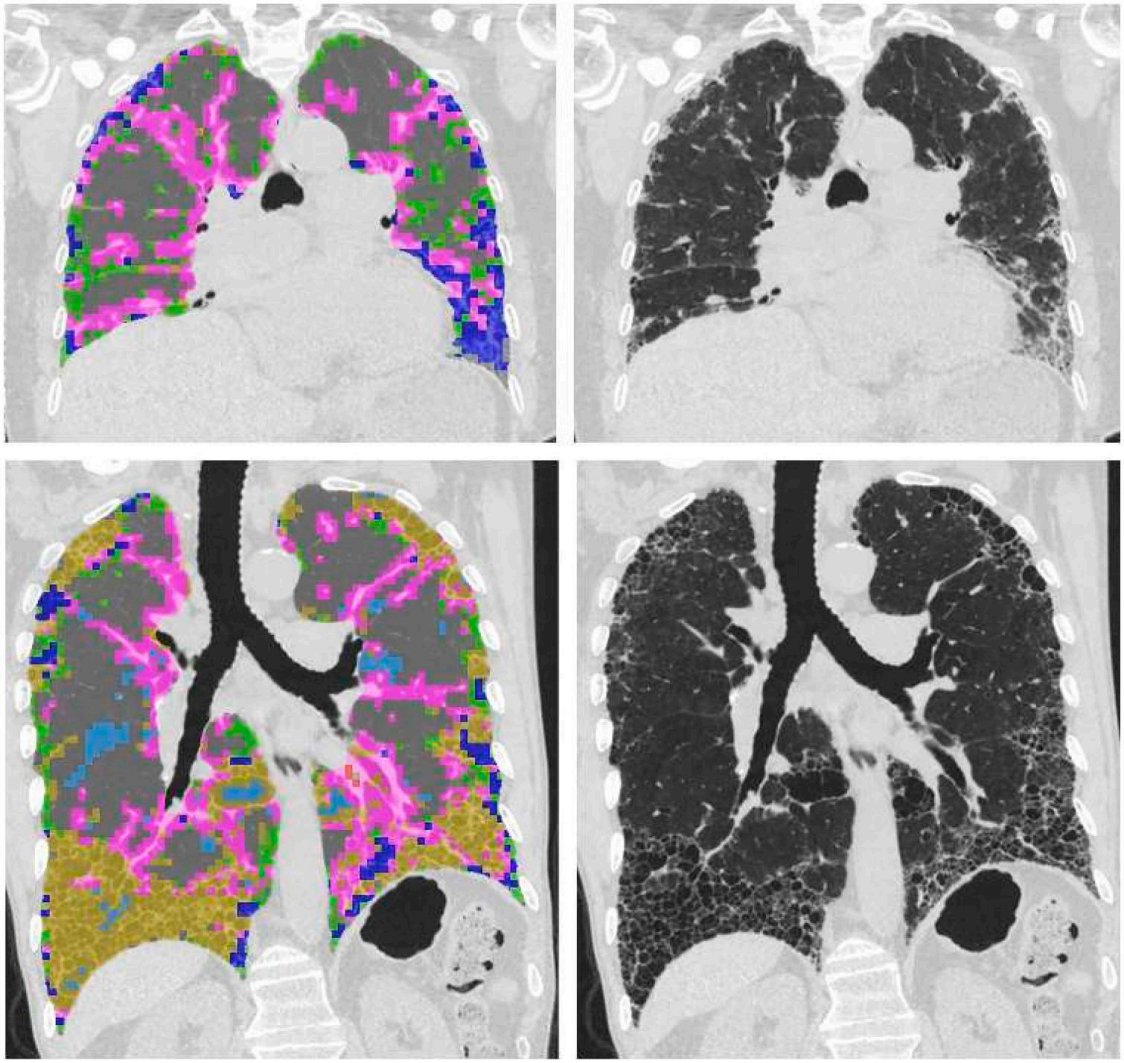
Adaptive multiple features method (AMFM). On the right, coronal reconstruction in a patient with IPF, depicting basal predominant fibrosis (coarse reticulation and honeycombing with traction bronchiectasis), characteristic of this disease. On the left, AMFM-based segmentation of the depicted parenchymal patterns [honeycombing (yellow), ground-glass opacities (blue), vascular structures (pink), and normal lung (grey)]. Courtesy of Prof. Eric Hoffman.

The adaptive multiple features method (AMFM) is an advanced HRCT analysis tool developed by the Department of Radiology at the University of Iowa.^6^ This tool utilizes mathematical representations of regional lung density, combined with a Bayesian classifier, to identify and quantify patterns of interstitial lung disease^21^^,^^22^ (Figure 1). The AMFM software was trained on 31 × 31 pixel image patches, each representing different HRCT features, and these patches were labelled by a consensus of expert radiologists. Early studies demonstrated promising levels of agreement between the AMFM tool and human observers in recognizing various interstitial pattern types, with a Cohen kappa statistic of approximately 0.62. A more recent sub-study involving patients enrolled in the PANTHER-IPF trial revealed that AMFM's quantification of baseline fibrosis and the assessment of fibrosis progression on HRCT independently correlated with disease progression and changes in forced vital capacity (FVC), respectively.^23^ These findings highlight the utility of the AMFM tool in tracking disease progression in patients with IPF.

### Functional respiratory imaging

**Figure 2.**
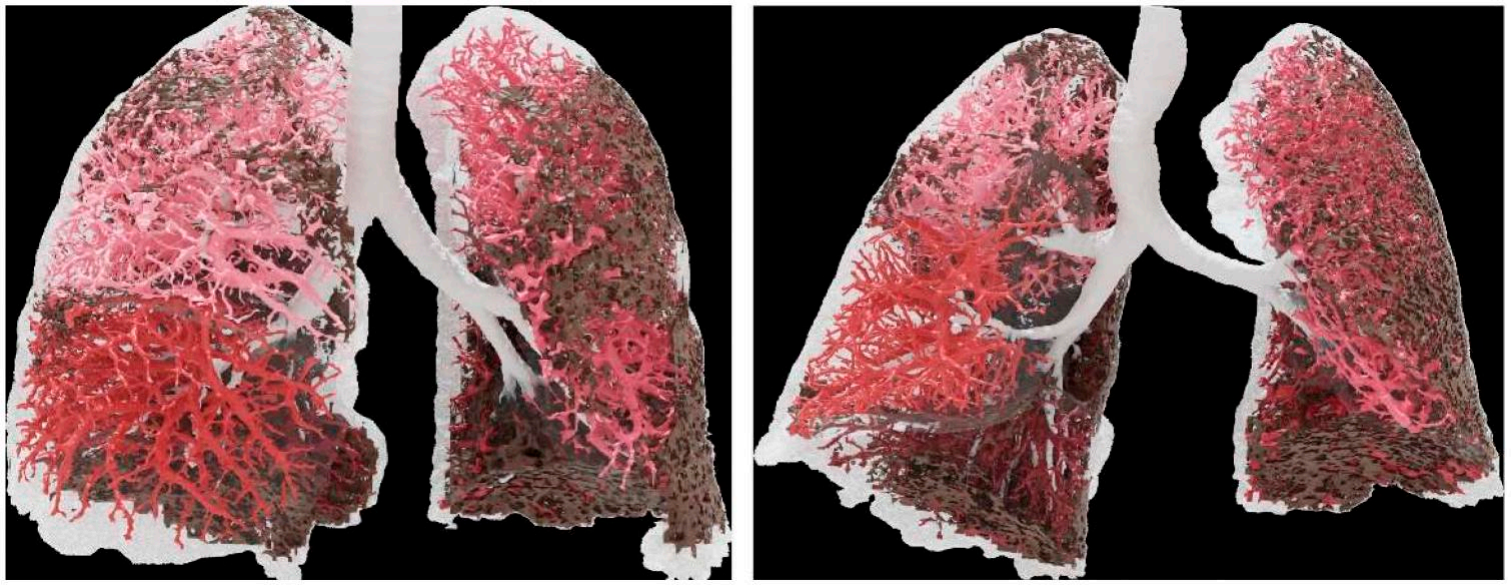
Functional respiratory imaging. Visualization and quantification of lobe volume, areas of fibrosis (depicted as dark areas), and blood vessel volume (structures in various shades of red). Courtesy of Dr. Jan De Backer.

Functional respiratory imaging (FRI), developed by Fluidda, combines low-dose high-resolution CT (HRCT) scans taken during inspiration and expiration with computer-based flow simulations. This data acquisition process involves respiratory gating using a handheld spirometer to ensure precise and repeatable lung volume measurements.^24^ FRI allows for the regional quantification of both lung structure and function and has been validated in obstructive lung diseases through comparisons with traditional lung function measurements, isotope-based techniques, hyperpolarized helium studies, exercise tolerance assessments, and patient-reported outcomes.^25^^,^^26^ Since FRI focuses on anatomical features such as airway and blood volumes, rather than individual voxels, it is less susceptible to measurement variability introduced by noise or reconstruction algorithms used in low-dose HRCT scan protocols^26^ (Figure 2).

Recent studies in patients with IPF have shown that disease progression, as indicated by declines in FVC, is associated with a reduction in HRCT-measured lung volumes.^27^ Changes in FVC are correlated with changes in lung volumes and changes in relative airway calibre. Also, disease progression in IPF is heterogeneous, with the lower lung lobes consistently more affected than the upper lobes. A key finding is that IPF progression, reflected by changes in FRI metrics, can affect lobe and airway volumes even when conventional measures like FVC remain within the normal range, suggesting that FRI may have utility in patients with marginal or subclinical disease. In a small drug trial using an autotaxin inhibitor, FVC showed a positive but non-significant trend, while FRI parameters confirmed a statistically significant treatment effect.^28^

## Deep learning

Traditional qCT tools are not without drawbacks. Many of the qCT tools described above rely on “feature engineering,” where the computer quantifies predefined CT patterns after training by expert radiologists. This approach has three key limitations. Firstly, the subjectivity of CT interpretation can affect the training process's reliability. Secondly, it assumes prior knowledge of the CT features reflecting disease extent, progression, or treatment response, potentially missing clinically significant but visually imperceptible patterns. Lastly, it necessitates manual image labelling, which is time-consuming and requires specialist radiologic expertise. These challenges can be overcome by using deep learning, which automatically learns important image features without the need for explicit feature labelling.

Deep learning is a branch of machine learning that is particularly efficient at recognizing patterns in complex data, such as the voxel data found in medical images and mapping these patterns to simple outcomes like diagnosis or future disease progression.^2^^,^^29–32^ While not a new concept, deep learning has its origins in the 1940s and 1950s with the development of computational algorithms called artificial neural networks (ANNs). Advances in computation have led to the development of more specialized algorithms optimized for image classification such as convolutional neural networks (CNNs) which have become the focus of intense research particularly in medical imaging. In respiratory medicine, high-accuracy CNNs have proven successful in applications like lung cancer detection, predicting mortality in COPD patients, and classifying fibrotic lung disease using CT scans.

The key feature of deep learning is that the training process is autonomous; it automatically identifies key features in CT scans for a given classification problem while ignoring irrelevant variations. This approach avoids the need for *a priori* knowledge of the most predictive image features and avoids time-consuming image annotation. It also allows for the possibility of discovering new CT features which are inaccessible visually. Techniques like attention mapping identify regions contributing to the algorithm's output, and dimensionality reduction methods provide insights into how the algorithm classifies disease.^2^^,^^29^^,^^33^ This capacity to uncover insights and enhance image classification makes deep learning a valuable tool in medical image analysis although arguably, progress developing methods for explaining deep learning algorithm outputs in fibrotic lung disease has not scaled with algorithm development.

### Deep learning and automated diagnostic support

Accurate radiologic diagnosis is essential in diagnosing fibrotic interstitial lung diseases, especially IPF. SLB decisions often hinge on precise HRCT IPF guideline-based classification and a typical UIP pattern on CT may obviate the need for SLB.^34^ Accurate CT interpretation also significantly influences patient management and impacts clinical trial enrolment criteria. However, application of HRCT guideline criteria is, like semi-quantitative evaluation, liable to significant interobserver variability, even among experts. This inconsistency in interpretation presents an opportunity for automated imaging-based decision support systems, demonstrated by a study reporting expert-level HRCT interpretation by a deep learning algorithm applying IPF HRCT guideline criteria.^2^^,^^6–8^ This technology could be cost-effective, particularly in regions with limited access to radiology expertise, and could improve patient stratification in clinical trials, reducing screen failures and associated costs.

### Early detection of progressive fibrotic lung disease

Interstitial lung abnormalities (ILAs) pose a challenging clinical issue. Current data from longitudinal cardiovascular and lung cancer screening cohorts suggest shared clinical and genetic links with IPF. ILAs are more common in older individuals, particularly smokers over the age of 50 who express the MUC5B promoter polymorphism.^35–37^ ILA occurs in about 7-9% of screened patients, however only a small minority of these represent subclinical progressive fibrotic lung disease.^38^ The key challenge is that it is currently not possible to predict which ILA are likely to progress.

This is a pattern recognition problem potentially amenable to deep learning-based solutions. However, two obstacles to deep learning-based ILA research exist. The first is that the current ILA classification relies on visually defined morphology and is broad, encompassing any CT pattern exceeding 5% in any lung zone. To enable algorithm training, an objective disease behaviour-based classification is likely to be needed.^39^ The second challenge is that progressive ILA represents only a small proportion of ILA overall meaning that data available on progressive ILA is sparse.^35^ Collation of longitudinal datasets by initiatives such as The Open-Source Imaging Consortium and collaboration between lung cancer screening programs will be needed to power this research. Cohort enrichment, possibly using serum biomarkers, will also be necessary for effective algorithm training.

### Predicting progressive pulmonary fibrosis

When fibrotic lung disease is established, broadly patients can progress despite treatment, be stabilized with treatment or remain stable without treatment. The term progressive pulmonary fibrosis (PPF) encompasses the first group of patients whose disease advances despite therapy and regardless of their clinical or histospecific diagnosis.^2^^,^^4^^,^^40–42^ Several positive clinical trials of antifibrotic therapy in non-IPF fibrotic lung diseases have focused on PPF, including the INBUILD study.^3^^,^^43^^,^^44^

One significant challenge in PPF lies in the lack of reliable methods to predict the progression of fibrotic diseases based on a patients’ baseline clinical and imaging data. Detecting PFF early would enable healthcare providers to initiate treatments at the earliest possible stage, without having to wait for clinical signs of progression or withhold treatment when the disease is inherently stable. Broad distinctions can be made between IPF and non-IPF disorders and between UIP-like and non-UIP-like diseases, but these categorizations are imprecise; non-UIP disease can be progressive while some patients with UIP may progress slowly. Extensive fibrosis on baseline CT is associated with an increased risk of progression.^3^^,^^44^ However, identifying PPF is particularly challenging when the baseline disease extent is less severe.

Like in ILA, identifying patterns of disease in CT that are likely to progress using baseline imaging may be possible using deep learning-based approaches although it will likely require the integration of other clinical data. As previously discussed, this approach allows for the possibility of identifying novel CT biomarkers of PPF that are imperceptible to the human eye. In principle, novel CT phenotypes that stratify patients with the same histospecific diagnosis into different outcome-based groups could be developed.^45^

### Deep learning-based CT phenotyping in fibrotic lung disease

One example of an emerging deep learning algorithm related to the detection of lung disease is SOFIA (systematic objective fibrotic imaging analysis algorithm). SOFIA identifies usual interstitial pneumonia and provides a “UIP probability” score which is a predictor of progression. This is an example of a novel biomarker predicting mortality regardless of disease severity, where conclusions were not drawn from HRCT alone, the UIP probability helped. A UIP probability can also made by radiologists, but generally radiologists will tend toward the extremes of the scale—either 0% or 100%, whereas SOFIA can provide a more accurate and detailed granular score, free from the biases and error that human assessment suffers from.^2^ Similar algorithm trained using multiple instance learning has demonstrated impressive classification accuracy using current IPF HRCT guideline criteria.^46^

An unsolved problem with SOFIA and similar deep neural networks is its interpretability; in cases where expert opinion and SOFIA-based outputs diverge, decoding algorithmic decision-making becomes critically important. This is one key challenge that the use of deep learning faces; the complexity of the neural network enables efficient high performance; however, this complexity also makes results difficult to interpret. Interpretability is also especially important in understanding an algorithm when it makes mistakes (e.g. misclassification of certain images).

### Deep learning-based qCT

#### Data driven textural analysis

**Figure 3.**
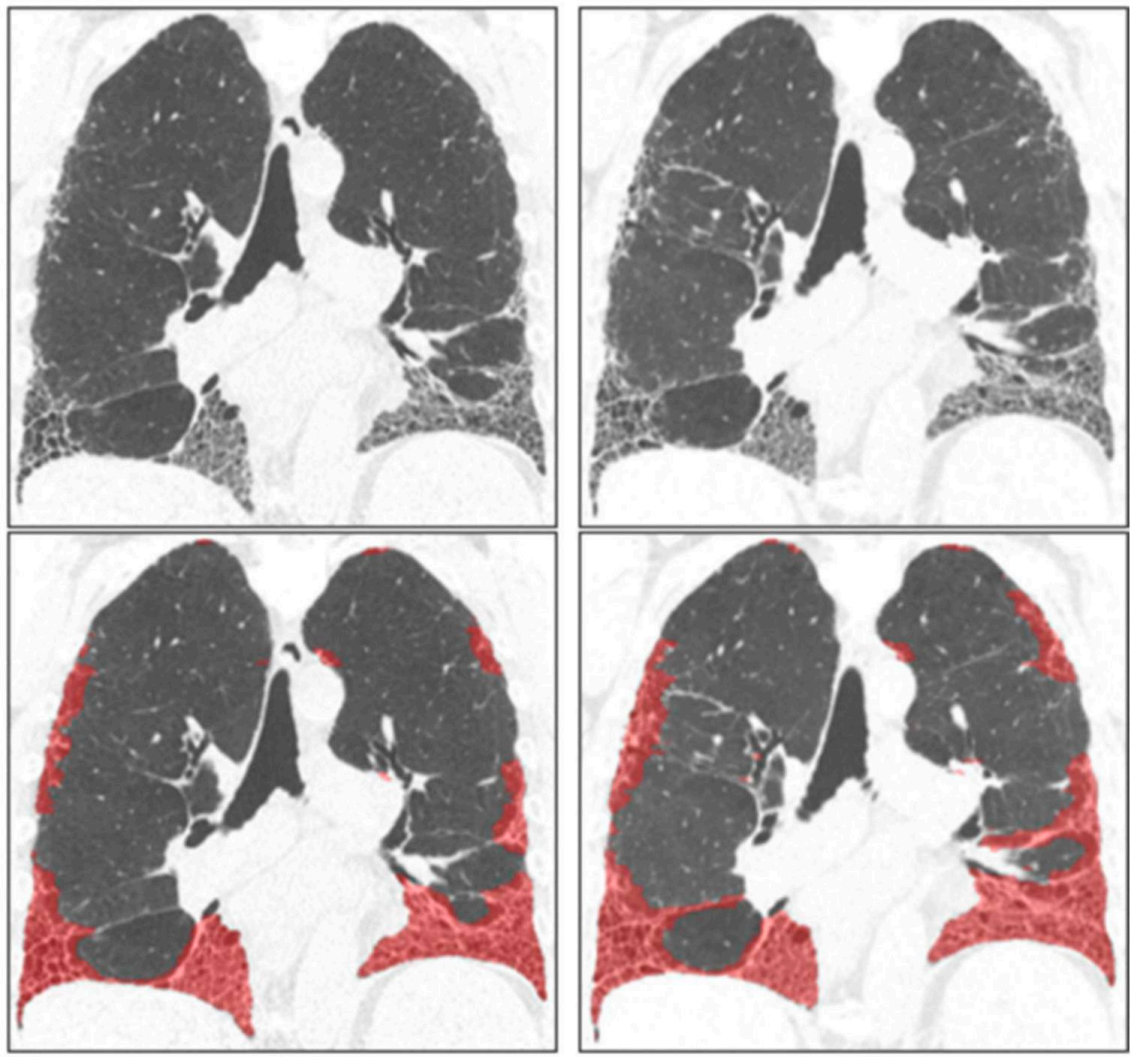
Data-driven texture analysis (DTA) in a patient with IPF at two timepoints. Top left panel is a coronal HRCT reconstruction depicting lower lobe honeycombing and reticulation which is quantified using DTA analysis (red overlay, bottom left panel), baseline FVC was 4.24 L (90.3% predicted), DTA score was 29.0%. On right (upper and lower panels) are coronal HRCT reconstructions in the same patient, 17 months later demonstrating progression of fibrosis. FVC was 4.01 L (86.1% predicted) and DTA score was 39.4%. Courtesy of Prof. Steve Humphries.

Data-driven texture analysis (DTA) classifies image segments based on the presence of fibrosis and subsequently quantifies the extent of this fibrosis across the HRCT, using a CNN. DTA generates a fibrosis score, which has shown to correlate well with lung function as well as the visual quantification of fibrosis by radiologists, and this score can be used to describe the extent of IPF (Figure 3). DTA has also successfully been used to aid in the prediction of disease progression, thanks to the information that the DTA score provides.^47–49^

#### Weighted vascular reticular scores

**Figure 4.**
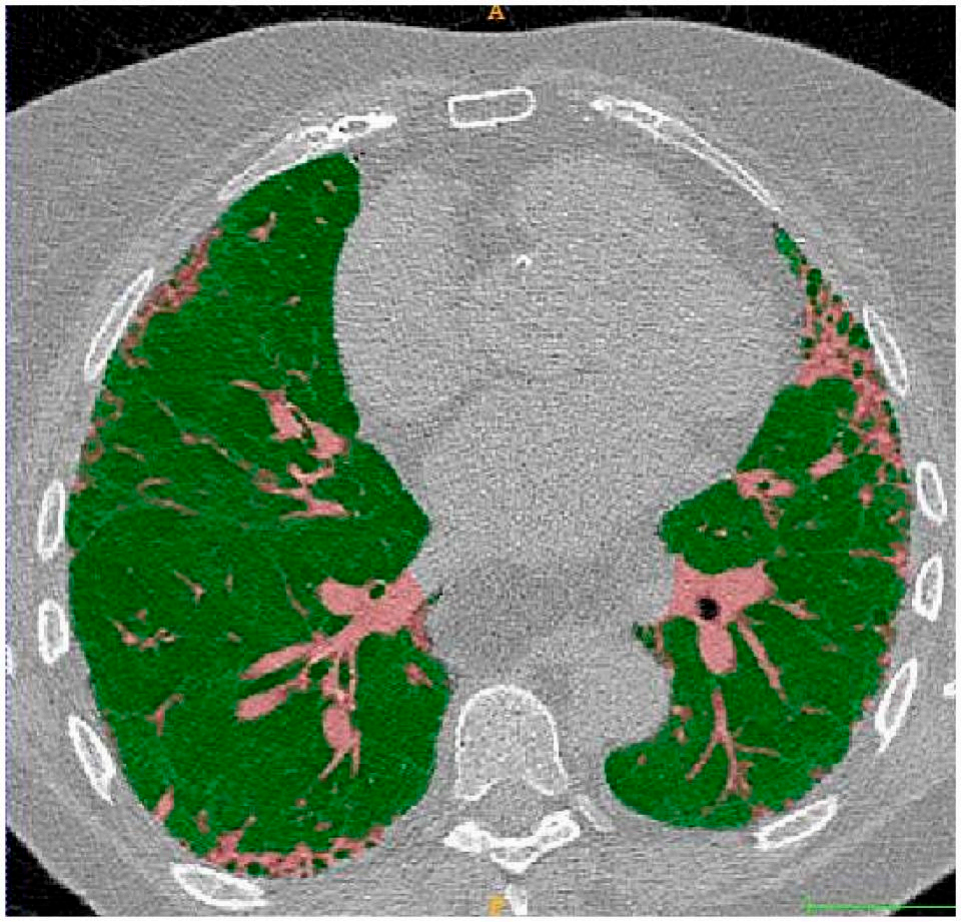
Brainomix. Axial HRCT in the lung parenchyma window, displaying the “weighted vascular-reticular score” (see text for details). Normal lung tissue is depicted in green, while vascular and reticular structures are segmented and quantified (pink). Courtesy of Dr. Peter George.

E-Lung is a deep learning-based tool developed by Brainomix, which quantifies reticular and vascular components of the lung and combines them as a single metric, the weighted vascular-reticular score (WRVS), for assessing disease severity in fibrotic lung disease (Figure 4). Early work reports that the WVRS may provide superior prognostic discrimination than traditional measures of disease severity, such as FVC. In a recent study of 352 non-IPF patients from The Open-Source Imaging Consortium, baseline WRVS score was a stronger predictor of transplant-free survival than FVC, both within individual cohorts and when pooling the data. Additionally, the WRVS was more predictive of future FVC decline over 12 months compared to baseline FVC.^50^^,^^51^ An exploratory clinical trial in IPF is underway to validate the use of the WVRS in conjunction with FVC for enhanced patient stratification and in principle, this novel metric could be used to monitor response to treatment in patients with fibrotic lung disorders.

#### Lung8, Air8, Vascul8, and Fibr8

**Figure 5.**
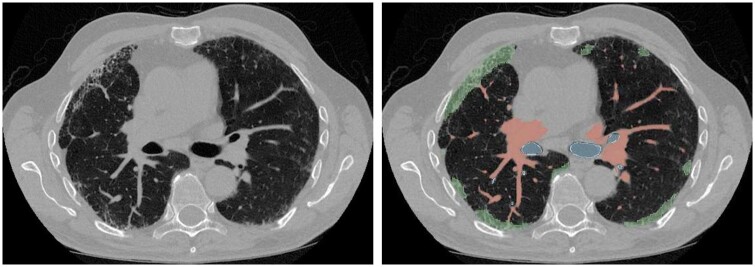
Qureight. Axial HRCT in the lung parenchyma window at a level below the carina, depicting deep learning-based segmentation provided by the Qureight deep learning platform. On the right are the segmented overlays: fibr8 (fibrosis extent score, green), air8 (airway assessment, blue), and vascul8 (pulmonary vessel volume assessment, pink). Courtesy of Dr. Muhuntan Thillai.

Qureight has developed four deep learning-based qCT algorithms which have been used to perform quantify specific CT-based biomarkers [volumes of the lung (Lung8), the airways (Air8), the pulmonary vasculature (Vascul8), and regions of fibrosis Fibr8] (Figure 5). These metrics have been used in several IPF studies, as *post hoc* analyses and as exploratory endpoints. For example, a significant reduction in CT-derived lung volume decline was shown versus placebo in the PINTA trial (NCT03725852).^52^ Airway volume was found to correlate with IPF disease progression,^53^ and an association with mortality has been demonstrated for both the automated fibrosis and vasculature volumes within a treatment naïve cohort from the PROFILE study.^54^^,^^55^

#### AIQCT

AIQCT is a deep learning-based qCT model for predicting the prognosis of IPF.^56^ The software was trained using 304 high-resolution computed tomographic (HRCT) scans from patients with various lung diseases and was designed to automatically identify and quantify 10 types of lung CT patterns and the airway volume, expressing these metrics as percentages of total lung volume. To validate AIQCT, its output was compared to visual scores from 30 HRCT images with lung diseases. AIQCT was then evaluated in 120 IPF patients: airway volume and lung volume, as measured by AIQCT, were independently associated with survival, adjusting for GAP stage.^57^ The conclusion highlights that AIQCT successfully quantified lung lesions and airway volumes and can provide additional prognostic information for IPF beyond traditional GAP physiology staging.

### Deep learning challenges

While advanced computer-based image analysis and in particular, deep learning offers huge potential in providing diagnosis support and disease quantification in fibrotic lung disease, it also comes with significant challenges relating to availability of training data, data and code sharing among stakeholders, mitigation of data biases, standardization of CT data, and ethical implementation.

Deep learning requires large fibrotic lung disease-specific imaging datasets for training, testing, and comparison, making establishing centralized imaging and clinical data repositories crucial. Diversity in data is particularly important in fibrotic lung diseases CT imaging protocols and scanner variations. Also, historically, collaboration among researchers in this field has been limited. Studies comparing various quantitative CT (qCT) tools using common imaging datasets are rare, partly due to competition and intellectual property concerns. Initiatives such as The Open-Source Imaging Consortium (OSIC; www.osicild.org), founded in 2018, address these issues by providing a diverse repository of CT chest images and clinical data from fibrotic lung disease patients and increasing research collaborations between academia and industry through machine learning competitions focused on digital biomarker development.

Deep learning algorithms also introduce unique risks, as they can amplify biases in training data and are susceptible to data manipulation.^58^ Issues like missing or unbalanced data not only affect algorithm performance but can also reinforce disparities in healthcare, particularly in rare disorders where inadequate training data can exclude patients from benefiting from this technology. Also, algorithm development can be manipulated to bias recommendations, favouring certain actions or third-party providers.^59^

Establishing ethical frameworks that foster trust and transparency is crucial for implementing artificial intelligence in healthcare. These frameworks should include bespoke data governance and accountability measures to ensure transparency in algorithm development and guidelines for data ownership, privacy, and sharing.^60^ Striking a balance between appropriate regulation and facilitating rapid innovation is not straightforward. The opacity of neural networks, the foundation of deep learning, is often seen as a drawback, known as the “black box phenomenon”.^61^ This complexity, while enabling pattern recognition in large datasets, can obscure reasoning, especially in medical applications where algorithms may rely on identifying and quantifying image features that are imperceptible visually. To integrate deep learning imaging biomarkers into clinical practice, better visualization methods are needed to assess their biological plausibility.

## Future directions

The application of AI in healthcare requires close collaboration between the AI community and clinical medicine; although deep learning can be effectively applied to medical imaging classification, the challenge lies in identifying clinical problems that need solving and developing tools that can truly impact clinical practice. As discussed, in some instances, improving algorithm interpretability will be key. For this reason, arguably, deep learning research in fibrotic lung disease should focus on quantifying interpretable variables, such as changes in fibrosis extent on CT over time or in response to therapy. As algorithm interpretability improves, novel image-based biomarkers including computer-generated radiological phenotypes responsive to specific therapies, might be used, facilitating precision medicine.

The mismatch between current technology and the availability of large, disease-specific imaging datasets for deep learning research in fibrotic lung diseases also needs to be addressed. This challenge, especially evident in cases of low-prevalence CT abnormalities that are likely to be important, such as ILA, can only be overcome by aggregating imaging datasets from various centres, driven by the mutual benefits of collaborative data sharing.

Finally, replacing radiologists with AI technology soon is unlikely. Instead, advances in computational image analysis, particularly in the era of deep learning, will enhance our understanding of and ability to interpret CT abnormalities in fibrotic lung disease. Collaboration between humans and machines, as suggested by Gary Kasparov (to quote, “a good human and a machine is the best combination”) is more likely to yield the best outcomes. Clear identification of AI research areas in medicine and integration with data from other domains will be vital to maximize the benefits of this synergistic relationship.

## Conclusion

Imaging-based biomarker research in fibrotic lung diseases is progressing rapidly, primarily due to advancements in image processing and analysis technologies. At the heart of this progress is machine learning, including deep learning. This technology has the potential to address key unmet needs in fibrotic lung disease such as detecting and categorizing ILA based on disease behavior, using baseline imaging to predict outcome in established fibrotic lung disease, and aiding precision medicine through better monitoring of therapeutic responses. Important enablers of these technologies are: the creation of precise clinical outcome definitions to aid in labeling training data, enhancing the interpretability of algorithms, building a body of evidence to show advantages over existing standards of care, and promoting regulations that facilitate the swift adoption of high-performing algorithms into everyday clinical practice. Eventually, digital biomarkers are expected to be further refined by integrating them with physiological, proteomic, and genomic biomarkers, maximizing the patient benefit.
